# The Role of Irisin in Alzheimer’s Disease

**DOI:** 10.3390/jcm7110407

**Published:** 2018-11-01

**Authors:** Oh Yoen Kim, Juhyun Song

**Affiliations:** 1 Department of Food Science and Nutrition, Dong A University, Busan 49315, Korea; oykim@dau.ac.kr; 2 Center for Silver-targeted Biomaterials, Brain Busan 21 Plus Program, Dong A University, Busan 49315, Korea; 3 Human Life Research Center, Dong A University, Busan 49315, Korea; 4 Department of Anatomy, Chonnam National University Medical School, Gwangju 61469, Korea

**Keywords:** irisin, Alzheimer’s disease (AD), glucose metabolism, insulin resistance, neurogenesis

## Abstract

Alzheimer’s disease (AD) is characterized by progressive memory dysfunction, oxidative stress, and presence of senile plaques formed by amyloid beta (Aβ) accumulation in the brain. AD is one of the most important causes of morbidity and mortality worldwide. AD has a variety of risk factors, including environmental factors, metabolic dysfunction, and genetic background. Recent research has highlighted the relationship between AD and systemic metabolic changes such as glucose and lipid imbalance and insulin resistance. Irisin, a myokine closely linked to exercise, has been associated with glucose metabolism, insulin sensitivity, and fat browning. Recent studies have suggested that irisin is involved in the process in central nervous system (CNS) such as neurogenesis and has reported the effects of irisin on AD as one of the neurodegenerative disease. Here, we review the roles of irisin with respect to AD and suggest that irisin highlight therapeutic important roles in AD. Thus, we propose that irisin could be a potential future target for ameliorating AD pathology and preventing AD onset.

## 1. Introduction

Alzheimer’s disease (AD), which is characterized by the accumulation of amyloid beta (Aβ) plaques, neurofibrillary tangles (NFT), and oxidative stress in the brain, is the most common subtype of dementia [1]. The risk factors related with the onset and development of AD are diverse and include genetic factors, environmental factors, and impaired metabolic activity [2,3,4]. Recent studies have reported that many metabolic disorders such as obesity, type 2 diabetes (T2DM), atherosclerosis, and cardiovascular disease, have been observed in patients with AD [5,6]. Considering previous evidences, the risk factors for AD are not limited to the central nervous system (CNS) and include systemic metabolic abnormalities. Irisin, a myokine that is formed by fibronectin type III domain containing protein 5 (FNDC5, aka PeP, and FRCP2) [7,8,9] and produced by muscle tissue [10] increases energy metabolism [8], regulates glucose homeostasis [11], and is directly related to the browning process, converting white adipose tissue into brown adipose tissue [11]. Moreover, recent studies have highlighted that irisin enhances brain function by modulating neurotransmitter secretion [12,13], and could play a beneficial role in the AD brain [14,15]. Considering the significant evidences that suggest that AD onset is related to metabolic changes, we hypothesize that irisin which is involved in various metabolic pathways, may be linked to multiple aspects of AD pathology. Here, we broadly review the possible effect of irisin on AD pathology and propose that the roles of irisin in CNS should be further investigated as a means of alleviating AD pathology.

## 2. Alzheimer’s Disease (AD)

AD is the most common subtype of dementia and ultimately leads to death [16,17,18]. Its pathologies result from Aβ plaques, which are formed by the proteolysis of Aβ precursor protein (APP) by β- and γ-secretases, and the accumulation of intraneuronal amyloid plaques [1]. Aβ oligomer uptake are associated with the endocytosis of N-methyl-D aspartate receptor and α-amino-3-hydroxy-5-methyl-4-isoxazolepropionic acid receptors [19,20], and is regulated by binding integrins [21] and are negatively related with the reduction of cellular cholesterol and sphingolipid levels [22]. Many studies suggested that decreased clearance process of Aβ in CNS leads to the development of AD [23]. The reduction of Aβ clearance process triggers the excessive accumulation of Aβ in the brain and cerebral amyloid angiopathy [24]. AD is characterized by cognitive impairment and memory loss [18], which are related to impaired synaptic plasticity and reduced long-term potentiation (LTP) [25]. Hippocampal neurogenesis plays a key role in synaptic plasticity and, ultimately influences learning and memory processes in the brain [26]. Patients with AD and AD animal models exhibit abnormal hippocampal neurogenesis [27,28,29]. The onset and development of AD are associated with genetic factors, mitochondrial dysfunction, oxidative damage, environmental factors, and energy metabolism impairment [2,3,4]. Clinically, the patients with AD typically have one or more comorbidity such as hypertension, obesity, diabetes, atherosclerosis, and metabolic deficiencies [5,6,30]. Most patients with the sporadic form AD are related to many pathogenic mechanisms, including brain atrophy, inflammation, oxidative stress, impaired glucose metabolism, insulin resistance, and T2DM [31,32,33]. As a result of findings from previous studies, recent research has highlighted the relationship between metabolic problems and AD [34,35]. One in vivo study has demonstrated that a high fat diet increases APP expression and APP processing enzyme levels [36]. Another in vivo study has reported that high fat diet fed rats exhibited increased levels of the APP-cleaving enzyme and Aβ in the hippocampus [37]. Furthermore, other studies have shown that a diet-induced obesity model exhibits memory dysfunction and neuroinflammation [35,38,39]. Moreover, the elevation of inflammatory responses in blood vessels triggers the reduction of synaptic plasticity and impairs neurogenesis [40]. Clinical studies have demonstrated that obesity results in decreased cognitive function, white matter atrophy, blood brain barrier disruption, and increases the risk of AD [41,42,43]. Even though the prevalence of AD is gradually increasing worldwide, effective therapeutic solutions and prevention methods are not fully found. As mentioned above, recent evidence has indicated a need for studying the relationship between AD onset and metabolic factors. Given the strong association between AD and metabolic changes, the study of metabolic problems in AD is necessary for finding therapeutic solutions for AD. 

## 3. What Is Irisin?

Irisin as a 112-amino acid glycosylated protein-hormone is formed by the proteolytic cleavage of FNDC5 [7,8,9]. Briefly, the polypeptide is proteolytically cleaved from the C-terminal moiety after the N-terminal signal peptide is removed, then glycosylated, and released as a hormone of 112 amino acids [7,8,9]. The production of irisin is similar with that of other hormones and hormone-like polypeptides (i.e., transforming growth factor-α, and epidermal growth factor from transmembrane precursors) [7,8,9]. The formation of FNDC5 is promoted by exercise in muscle, and it could be changed into irisin by the transcriptional co-activator PPAR-*γ* co-activator-1 *α* (PGC1*α*) [8]. Irisin is secreted by muscle, circulates in the fat tissue and regulates energy metabolism [10,44,45]. It is known as the cold induced endocrine regulator of brown fat function [11,46] and its secretion is also promoted by the cold exposure [11,47]. In humans, mRNA expression of *FNDC5* is approximately 100 times lower in the adipose tissue than in the skeletal muscle [48,49,50]. Secretion of irisin from adipocytes is also lower than that from skeletal muscle [49,50]. The irisin level in human white adipose tissue is only 5% of the level found in skeletal muscle [51]. Additionally, irisin, both from peripheral and neuronal origins, circulates throughout the body [52]. Mammals have two types of adipose tissue, white and brown [53]. Irisin promotes the browning process of white adipose tissue into brown adipose tissue [8,11,54,55]. The excess accumulation of white adipose tissue leads to obesity and results in an energy imbalance and chronic inflammation [56,57]. Brown adipose tissue is responsible for energy thermogenesis [58,59,60] due to the presence of many large mitochondria [61,62]. Irisin stimulates fat browning by activating mitogen-activated protein kinase p38 MAP kinase and ERK MAP kinase signaling [63]. Furthermore, the secretion of irisin has been reported to increase after exercise [64,65] and influence the regulation of glucose homeostasis and lipid metabolism in skeletal muscle and adipose tissue [66,67]. Several studies have demonstrated that irisin, a contraction-regulated myokine, is secreted during exercise and exerts beneficial effects on glucose homeostasis [11,68]. In vivo studies have shown irisin injection to lead to an increase in energy expenditure and improvement in glucose metabolism [8,49,69,70]. Moreover, irisin is known to play crucial roles in the CNS [45]. Previous studies have reported that irisin is observed in the cerebrospinal fluid (CSF) and hypothalamus [71], and *FNDC5* is known to be highly expressed in glia (e.g., astrocytes and microglia) and neurons in various brain regions [72,73,74,75]. Irisin is synthesized in the muscle tissue and is present in cerebellar Purkinje cells and intercellular nerve endings [73,76]. Zhang et al. demonstrated that administration of irisin by intracerebroventricular injection leads to an increase of the locomotor activity [77]. According to Brailoiu et al., irisin promotes neuronal depolarization of cardiac projecting neuron nucleus [78]. One study suggested that irisin contributes to neural differentiation by modulating metabolic responses in the CNS [77]. In rodents, FNDC5 is found in several brain regions, such as the midbrain and the hippocampus [79], and is known to be involved in reward-related models [80]. Irisin exerts antidepressant-like effects via modulation of energy metabolism in the prefrontal cortex [81]. A recent study demonstrated that skeletal muscle-derived irisin is linked to reward-related processes and motivation [82]. Several studies have highlighted that irisin secreted after exercise plays a beneficial role in brain function [12,13] and in neurodegenerative diseases such as AD [14,15]. Considering the multiple effects of irisin, we reviewed the various roles of irisin in the AD brain.

## 4. The Potential Therapeutic Roles of Irisin in Alzheimer’s Disease

### 4.1. Irisin Improves Learning and Memory Function by Regulating the Expression of Brain-Derived Neurotrophic Factor (BDNF)

Brain-derived neurotrophic factor (BDNF) as the widely distributed neurotrophin in the brain has been known to has a critical role in synaptic function and neuronal survival [83]. Also, BDNF suppresses the cytotoxic response of neuron and learning deficits against Aβ toxicity in AD [84,85]. Irisin is the upstream mediator of BDNF production [64] and triggers the expression of BDNF [64,86]. The exercise induced increase of FNDC5/irisin release from the periphery results in the increase of FNDC5/irisin in the neurons as well as the increase of BDNF production in the neuron [64]. They demonstrated that the manipulation of *FNDC5* expression by using siRNA in cortical neuron results in the decrease of *BDNF* expression [64]. BDNF controls dopamine-3 signaling pathways, influencing dopamine’s effect on the brain [87,88] and modulating synaptic plasticity and neurogenesis in the amygdala, prefrontal cortex, and hippocampus [86,89]. BDNF induces LTP and increases the strength of synaptic connections [90]. Yan et al. reports the connection between BDNF and mesocortico-limbic system in brain and also proves the importance of BDNF in brain circuits [90]. Furthermore, BDNF is necessary for hippocampal neurogenesis and the hippocampal neural circuit [91,92]. BDNF is also known to play an important role in LTP as well as learning and memory function [93,94]. Decreased levels of neurotrophins such as BDNF have been shown in various regions of the AD brain [95,96,97]. Several studies have demonstrated that the administration of BDNF in patients with AD ameliorates AD pathology [98,99]. Recent studies have demonstrated that the irisin-BDNF axis could strengthen learning and memory function [82,100,101] and improve mood disorders and anxiety [102,103,104]. FNDC5/irisin contributes to the boosting of reward-related learning and motivation by enhancing the production of BDNF [82]. In addition, the increased expression of *FNDC5* in primary cortical neurons promotes *BDNF* expression, while knockdown of *FNDC5* leads to a decrease in *BDNF expression* [105]. Given that the expression of *BDNF* could potentially improve cognitive decline in AD [99], the stimulation of the irisin-BDNF axis in the brain may be a promising issue for AD treatment.

### 4.2. Irisin Promotes Neurogenesis and Protects against the Neuronal Damage Caused by Oxidative Stress

Neurogenesis which mainly occurs in the subventricular zone and the dentate gyrus of the hippocampus is important in in structural synaptic plasticity and neural network maintenance and contributes to the recovery of cognitive dysfunction [106,107]. Several studies reported that the neurogenesis as the new neuron’s replacement process in learning related hippocampus region is attenuated in AD brain [108,109]. Irisin has been shown to be essential for neural differentiation in mouse embryonic stem cells [110,111]. Knockdown of *FNDC5* in neuronal precursor cells inhibits the differentiation of mouse embryonic stem cells into neurons, and the maturation of astrocytes [110]. In addition, FNDC5 boosts the differentiation of embryonic stem cells into neural cells [112]. One study found that irisin affects hippocampal neurogenesis and induces neuronal proliferation by modulating neurogenesis-related STAT3 signaling [111,113]. Huh et al. demonstrated that knockdown of the precursor of irisin suppresses the neural differentiation of embryonic stem cells in mice [48]. Irisin inhibits the neuronal damage caused by oxidative stress through activation of Akt/ERK1/2 signaling [114]. Irisin protects neurons by attenuating the secretion of pro-inflammatory cytokine such as tumor necrosis factor (TNF)-α through Akt/ERK1/2 signaling [114]. In addition, irisin protects neurons by suppressing ROS-NLRP3 inflammatory signaling in ischemic conditions [115]. This study demonstrated that PC12 cells as neuronal cells inhibited the expression of pro-inflammatory cytokine, interleukin (IL)-1β and the activation of caspase 1 signaling as apoptosis signaling under ischemic condition (i.e., oxygen and glucose deprivation conditions) [115]. Given that the enhancement of neurogenesis could potentially improve the impaired synaptic plasticity and memory dysfunction found in AD [26,28,29], irisin may be a potential therapeutic target for AD as it promotes both neurogenesis and neuronal cell survival.

### 4.3. Irisin Ameliorates Insulin Sensitivity and Improves Glucose Homeostasis

Impaired cerebral glucose metabolism and insulin signaling as a major pathological feature of AD is critically involved in the learning and memory loss [116]. The impairment of glucose uptake in the brain triggers brain atrophy and neuronal dysfunction in AD [117]. One study reported that glucose transporter (GLUT) 1 deficiency in brain endothelial cells aggravates AD neuropathology such as cognitive decline [118]. According to previous studies, irisin acts as a regulator of glucose utilization and browning of white adipose tissue in models of obesity and T2DM [63,119]. Serum irisin levels and single nucleotide polymorphisms in the *FNDC5* are associated with glucose metabolism and insulin resistance [120]. A clinical study reported that circulating irisin levels are higher in patients with metabolic syndrome than in normal subjects [121]. A reduction in circulating irisin levels has been associated with insulin resistance [122,123]. Several studies have demonstrated that irisin has beneficial effects on insulin sensitivity and glucose metabolism in both skeletal muscle and adipose tissue [66,67]. Serum irisin levels were founded significantly lower in diabetic subjects than in normal subjects [124]. Irisin decreases the expression of *G6Pase* and *PEPCK*, both of which are critical for gluconeogenesis, in the liver [66]. Furthermore, irisin controls glucose utilization via p38 MAPK signaling [63]. Previous studies reported the strong association between FNDC5/irisin and insulin resistance estimated by homeostatic model assessment [125], and by testing glucose tolerance [50]. A study assessed the insulin action by irisin through Akt activation [124]. FNDC5/irisin overexpression improves insulin resistance and reduces blood glucose in the high fat diet fed mouse [126]. Plasma membrane GLUT-4 expression is activated in the skeletal muscles of irisin-treated rats [63,119]. Based on the previous reports, we also hypothesize that irisin may be a central modulator of glucose metabolism and insulin activity in the AD brain.

## 5. Conclusions

**Figure 1:**
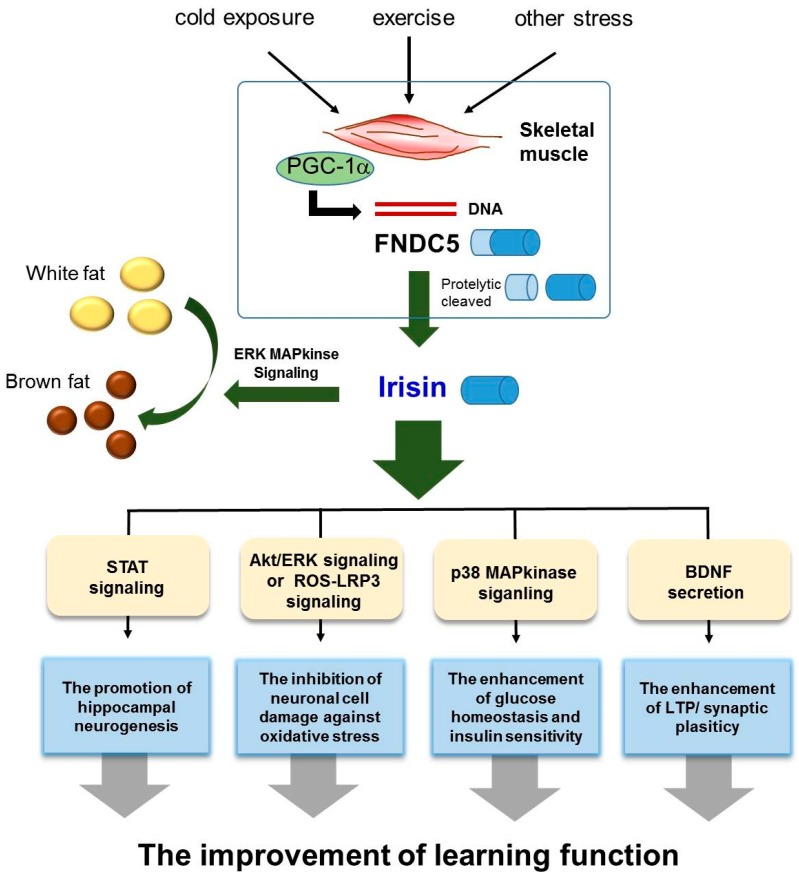
Scheme for the therapeutic role of irisin in Alzheimer’s disease.

We reviewed the role of irisin in the AD brain, and demonstrated that irisin is involved in the modulation of several AD risk factors, including insulin resistance, impaired neurogenesis, oxidative stress, and imbalance of neurotrophic factors. In this review, we highlighted three points concerning the potential therapeutic role of irisin in AD (Figure 1). First, irisin boosts the production of BDNF, which could subsequently lead to cognitive improvement and a reduction in synaptic dysfunction in AD. Second, irisin could potentially enhance neurogenesis and protect against neuronal damage in AD. Finally, irisin might have a role in the regulation of insulin resistance and glucose homeostasis in AD. It is necessary to find a suitable treatment for alleviating AD pathology, and irisin holds promise as a therapeutic hormone. For example, several studies have demonstrated that irisin can be referred to as an “exercise-hormone” [8] since induction of FNDC5 by endurance exercise [8] has been observed in mice [71,127] and humans [128]. In addition, exercise induced irisin expression in the hippocampus could activate BDNF and other neuroprotective genes [64]. Based on previous studies, the treatment of irisin could suppress the expression of pro-inflammatory cytokines such as TNF-alpha and IL-6, reduce the expression of monocyte chemoattractant protein-1 in adipocytes, subsequently attenuate migration of macrophages and induce the phenotypic switching of macrophages from M1 (pro-inflammatory) to M2 (anti-inflammatory) state [129]. Also, in brain ischemia state, the researchers observed that marked increased levels of ROS and malondialdehyde were reduced by irisin treatment in peri-infarct brain tissues [130]. Furthermore, uncoupling proteins, as a regulator in mitochondrial biogenesis and neuroprotection and synaptic function in the CNS, was increased by high dose administration of irisin in all brain areas [131,132,133]. Given these clinical application of irisin, we speculate that irisin may contribute to the improvement of neuropathy by reducing neuroinflammation, and enhancing synaptic functions in AD brain. In light of the presented evidence, we propose that AD patients require the induction of FNDC5 through an appropriate amount of exercise to promote neurogenesis, neuronal survival, and synaptic plasticity. Therefore, we propose that irisin could be a potential future target for ameliorating AD pathology and preventing AD onset.
